# Target-centered network pharmacology and molecular docking reveal potential mechanisms and bioactive compounds of Zhigancao Decoction in insomnia–Arrhythmia comorbidity

**DOI:** 10.1097/MD.0000000000049892

**Published:** 2026-07-31

**Authors:** Bolun Xue, Linghui Lu, Yong Wang, Wei Wang

**Affiliations:** a College of Traditional Chinese Medicine, Beijing University of Chinese Medicine, Beijing, 102488, China.

**Keywords:** Arrhythmia, Insomnia, Molecular docking, Network pharmacology, Renin–angiotensin system, Target-centered reverse screening, Traditional Chinese medicine, Zhigancao Decoction

## Abstract

This study aimed to investigate the potential mechanisms of Zhigancao Decoction (ZGCT) in insomnia–arrhythmia comorbidity using a target-centered network pharmacology and molecular docking approach. In addition, a refined candidate bioactive compound library was constructed. Active components of ZGCT were retrieved from TCMSP and HERB databases, and target prediction was performed using SwissTargetPrediction. Disease-related targets for insomnia and arrhythmia were obtained from GeneCards, followed by identification of intersection targets. A protein–protein interaction (PPI) network was constructed using STRING, and hub genes were identified via topological analysis and Maximal Clique Centrality (MCC) in Cytoscape. Functional enrichment analysis was performed using Metascape, and molecular docking was used to evaluate ligand–target interactions. A total of 84 active compounds and 193 associated targets were identified for ZGCT. Intersection analysis yielded 41 common targets. PPI network analysis identified 10 hub targets, including AKT1, PPARG, and MMP9. Based on a target-centered reverse screening strategy, 22 candidate bioactive compounds were identified, and molecular docking showed computationally favorable binding energies between key compounds and core targets. These compounds are associated with neuroendocrine, inflammatory, and cardiovascular signaling pathways. ZGCT is predicted to exert therapeutic effects on insomnia–arrhythmia comorbidity through a systems-level multi-target regulatory network involving neuroendocrine modulation, renin–angiotensin system-related pathways, and cardiovascular remodeling. The proposed target-centered reverse screening strategy provides a refined and interpretable framework for constructing a disease-specific bioactive compound library, offering potential directions for further experimental validation and drug development.

## 1. Introduction

Insomnia and arrhythmia are common clinical conditions that frequently coexist in clinical practice. Epidemiological studies have demonstrated a bidirectional association between sleep disturbances and cardiovascular diseases.^[1]^ A nationwide cohort study involving 193,263 participants reported that insomnia is significantly associated with an increased risk of atrial fibrillation (AF), particularly in males, individuals aged over 65 years, and patients with peripheral arterial disease.^[2]^ Furthermore, persistent insomnia has been identified as an independent predictor of long-term atrial fibrillation (AF) recurrence following radiofrequency ablation, with a dose–response relationship.^[3]^ The 2025 American Heart Association scientific statement on multidimensional sleep health underscores the critical role of sleep continuity and sleep quality in cardiometabolic health, highlighting sleep disorders as modifiable risk factors for cardiovascular disease.^[4]^ Despite this growing recognition, the pharmacological management of insomnia–arrhythmia comorbidity remains suboptimal, as conventional treatments often target a single condition and may exacerbate the other.

The pathophysiological interaction between insomnia and arrhythmia is complex and involves systemic inflammation, autonomic nervous system dysfunction, oxidative stress, and metabolic dysregulation.^[5]^ Patients with insomnia, particularly those with objectively short sleep duration, exhibit markedly increased hypothalamic–pituitary–adrenal (HPA) axis activity, accompanied by elevated secretion of cortisol and other stress-related hormones. Sustained HPA-axis activation and associated endocrine imbalance further increase the risk of metabolic syndrome and coronary heart disease.^[6]^ In addition, sleep deprivation promotes systemic inflammation, thereby contributing to cardiovascular pathogenesis.^[7]^ Poor sleep quality is also closely associated with oxidative stress, representing another key mechanism through which sleep disruption contributes to cardiovascular disease.^[6]^ Collectively, these converging pathological pathways suggest that therapeutic strategies targeting shared molecular nodes, rather than isolated symptom suppression, may provide superior efficacy for the management of comorbid conditions.

Current pharmacological strategies for insomnia–arrhythmia comorbidity remain largely fragmented and may even exert opposing effects. nonbenzodiazepine hypnotics, such as zolpidem, which are widely used for the treatment of insomnia, have been associated with an approximately two-fold increased risk of incident cardiac arrhythmias, including paroxysmal ventricular tachycardia and atrial fibrillation.^[8]^ Conversely, benzodiazepines may induce rebound insomnia and withdrawal symptoms. In addition, their muscle-relaxant properties may impair respiratory function during sleep, potentially worsening cardiovascular outcomes in susceptible individuals.^[9]^ This therapeutic paradox, in which treatment of one condition may aggravate the other^[10]^ highlights the urgent need for multi-target therapeutic strategies that can simultaneously address both sleep disturbances and cardiac rhythm abnormalities without inducing reciprocal adverse effects.^[11,12]^

Traditional Chinese Medicine (TCM) provides a distinctive theoretical framework for managing comorbid diseases through the concept of “Yibing Tongzhi” (treating different diseases with the same therapeutic principle),^[13,14]^ This concept proposes that different clinical conditions sharing a common pathogenesis can be treated using similar therapeutic strategies.^[15,16]^

Zhigancao Decoction (ZGCT) originates from Treatise on Cold Damage Diseases (Shang Han Lun), a classical medical text compiled by Zhang Zhongjing during the Eastern Han Dynasty in China. This formula is composed of honey-fried licorice root (Zhigancao), ginseng (Renshen), dried rehmannia root (Shengdihuang), donkey-hide gelatin (Ejiao), Ophiopogon japonicus (Maidong), hemp seed (Huomaren), cinnamon twig (Guizhi), fresh ginger (Shengjiang), and jujube (Dazao).

Clinical observations have shown that ZGCT demonstrates therapeutic potential in patients with insomnia accompanied by palpitations, improving sleep quality while alleviating palpitations^[17]^ These effects reflect the TCM principle of “treating different diseases with the same method” (Yibing Tongzhi).

In this study, network pharmacology and molecular docking approaches were employed^[18]^ to explore the multi-target regulatory mechanisms of ZGCT in insomnia–palpitation comorbidity. This work aims to construct a candidate compound library and identify potential molecular targets for further investigation of heart–brain-related comorbid disorders.^[19]^

## 2. Materials and methods

### 2.1. Screening of active ingredients and acquisition and prediction of potential targets

The Traditional Chinese Medicine Systems Pharmacology Database and Analysis Platform (TCMSP) (https://tcmsp.91medicine.cn/#/home) was used to retrieve the chemical constituents of Zhigancao Decoction (ZGCT), including Zhigancao, Renshen, Shengdihuang, Ejiao, Maidong, Huomaren, Guizhi, Shengjiang, and Dazao. Active compounds were screened using the criteria of oral bioavailability (OB) > 30% and drug-likeness (DL) ≥ 0.18,^[20]^ and compounds meeting these criteria were retained.

For herbal components not included in TCMSP, the HERB database (http://herb.ac.cn/) was used for supplementary retrieval. The identified compounds from both TCMSP and HERB databases, together with experimentally reported in vivo constituents^[21]^ and compounds documented in the Chinese Pharmacopoeia, were further processed.

All chemical structures were obtained from the PubChem database (https://pubchem.ncbi.nlm.nih.gov/) in SMILES or SDF format. These structures were subsequently submitted to the SwissTargetPrediction database (http://www.swisstargetprediction.ch/) for target prediction, with species restricted to Homo sapiens. Predicted targets with a probability score > 0.3^[22]^ were selected as potential bioactive compound-related targets.

### 2.2. Acquisition and screening of targets for insomnia and arrhythmia diseases

Disease-related targets associated with insomnia and cardiac arrhythmia were retrieved from the GeneCards database (https://www.genecards.org/) using the keywords “Insomnia” and “Cardiac Arrhythmia,” respectively. The retrieved target lists were subsequently exported for further analysis.

To improve data reliability, targets were filtered using the median score method provided by GeneCards. Specifically, only targets with a relevance score higher than the median value were retained for each disease.^[15]^ The filtered target sets for insomnia and cardiac arrhythmia were then intersected to identify shared targets potentially involved in the comorbidity.

Finally, a Venn diagram was generated using the online tool Venny 2.1.0 (https://bioinfogp.cnb.csic.es/tools/venny/) to visually represent the overlap between the 2 disease-related target sets and to facilitate subsequent network construction and functional analysis.

### 2.3. Construction of the “Drug Component - Target” network

The intersection targets identified from the active components of Zhigancao Decoction (ZGCT) and disease-related genes were organized into “attribute” and “network” files. These datasets were subsequently imported into Cytoscape 3.9.1 software to construct a drug component–target interaction network.

This network was used to visualize and analyze the complex relationships between bioactive compounds and shared therapeutic targets associated with insomnia and arrhythmia comorbidity, thereby facilitating the identification of key compound–target interactions within the pharmacological network.

### 2.4. Construction of protein-protein interaction networks and screening of core targets

Import the intersection targets into the STRING database (https://cn.string-db.org/), and set the species as *Homo sapiens*. The interaction confidence level was set to 0.9 (“highest confidence”),^[15]^ while all other parameters were kept at default settings. After removing isolated nodes, a protein–protein interaction (PPI) network was obtained.

The resulting network was imported into Cytoscape 3.9.1 software for visualization. Topological analysis was then performed, and core targets were identified using the Maximal Clique Centrality (MCC) algorithm implemented in the CytoHubba plugin.^[23]^ The top 10 nodes ranked by MCC scores were selected as hub genes for further analysis.

### 2.5. Functional enrichment analysis

The intersection targets were submitted to the Metascape online platform (https://metascape.org/gp/index.html) for functional enrichment analysis. The species was restricted to Homo sapiens, and “Custom Analysis” was selected.

Gene Ontology (GO) enrichment analysis was performed to evaluate biological process (BP), cellular component (CC), and molecular function (MF), respectively. In addition, Kyoto Encyclopedia of Genes and Genomes (KEGG) pathway enrichment analysis was conducted to identify significantly associated signaling pathways.

The enrichment results were subsequently visualized for further interpretation.

### 2.6. Molecular docking verification

Molecular docking was performed using a target-centered reverse screening strategy. Unlike traditional network pharmacology approaches that prioritize compounds based on topological parameters such as degree centrality in compound–target networks, the present study focused on biologically prioritized protein targets.

Specifically, the top 10 hub targets identified from the protein–protein interaction (PPI) network were selected as receptor proteins. In parallel, bioactive small molecules were obtained from SwissTargetPrediction based on structural similarity and probability scoring (Homo sapiens, *p* > .3), representing ligand candidates with predicted biological relevance to human protein targets.

To systematically evaluate ligand–target interactions, a full-matrix docking strategy was employed. Each of the 10 hub targets was docked against all SwissTargetPrediction-derived compounds, generating a comprehensive interaction matrix covering all possible ligand–target combinations.

Three-dimensional structures of protein targets were retrieved from the Protein Data Bank (PDB) (https://www.rcsb.org/), and ligand structures were obtained from the PubChem database (https://pubchem.ncbi.nlm.nih.gov/). Molecular docking was performed using CB-Dock2 (https://cadd.labshare.cn/cb-dock2/php/index.php), and binding affinity was evaluated using binding energy (kcal/mol). Lower binding energy values indicate more stable binding conformations.

## 3. Results

### 3.1. Screening results of drug active ingredients and disease targets

A total of 84 active components of Zhigancao Decoction (ZGCT) were identified from the TCMSP and HERB databases. These included 13 compounds from Dazao, 6 from Guizhi, 5 from Huomaren, 6 from Renshen, 4 from Shengjiang, 4 from Shengdihuang, and 33 from Zhigancao. In addition, 8 in vivo–identified components and 5 compounds documented in the Chinese Pharmacopoeia were also included. After integration and standardization, a total of 193 drug-related targets were obtained.

Disease-related targets were retrieved from the GeneCards database using “Insomnia” and “Cardiac Arrhythmia” as keywords. A total of 2346 targets for insomnia and 3685 targets for cardiac arrhythmia were identified.

**Figure 1.**
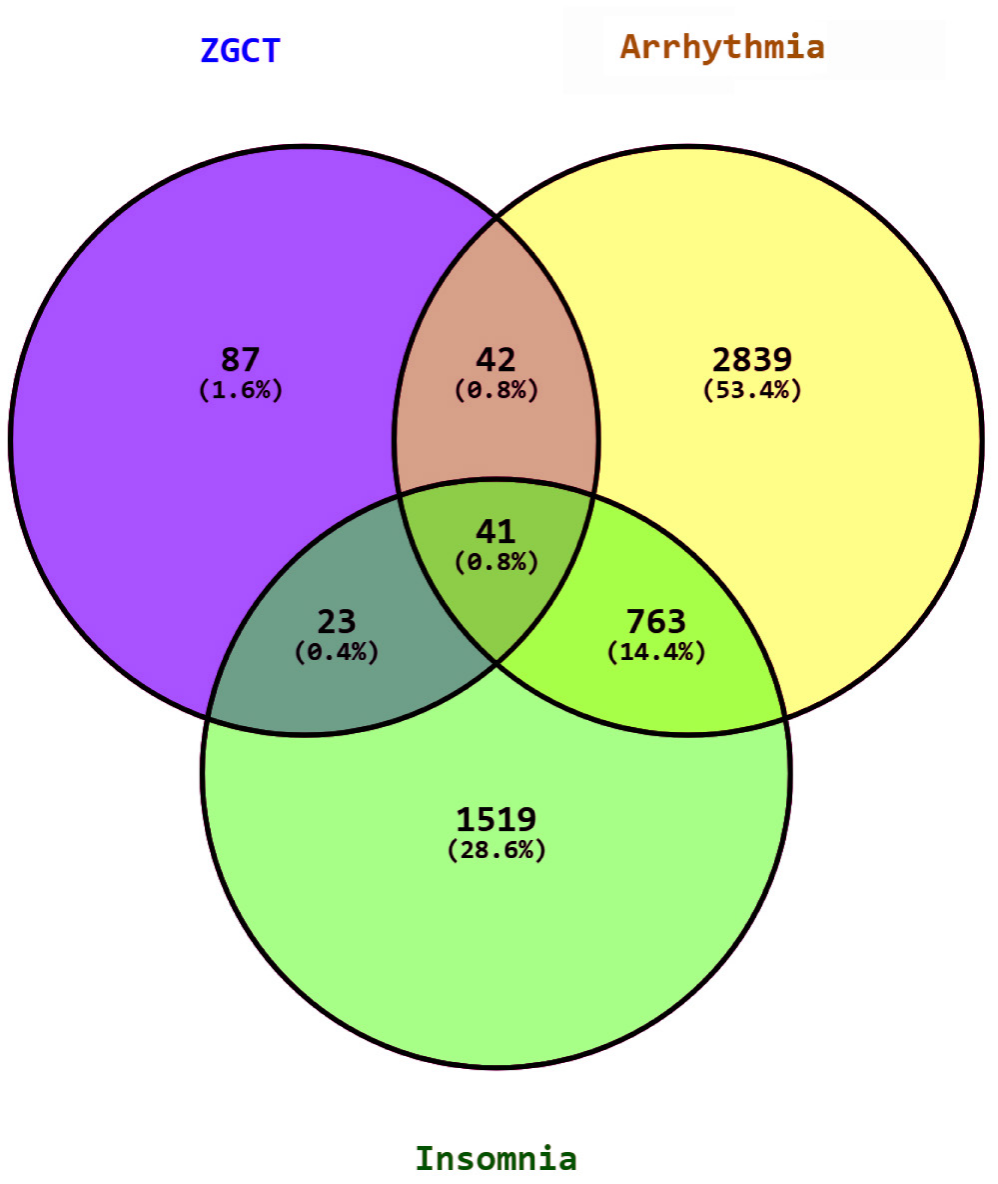
Venn diagram of Zhigancao Decoction and the 2 diseases. The overlapping parts indicate the number of target genes that are intersected.

The intersection between drug-related targets and disease-related targets yielded 41 common targets, as shown in Figure 1.

### 3.2. The construction results of the drug component - target network

**Figure 2.**
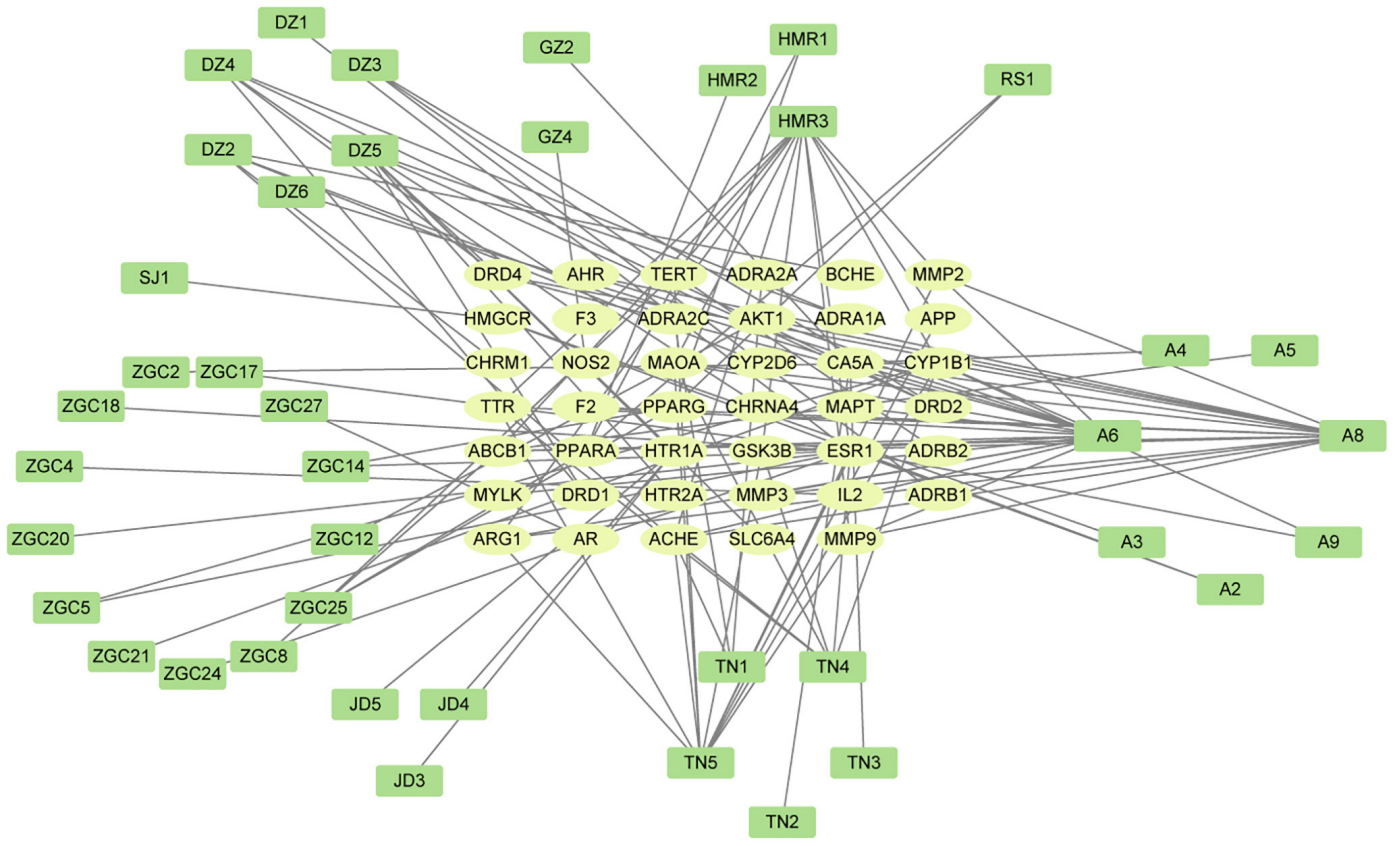
“Drug component - target” network diagram of the interaction points among Zhigancao Decoction, insomnia and arrhythmia.

Based on the above results, a drug component–target interaction network was constructed using Cytoscape 3.9.1 software, as shown in Figure 2.

The results indicated that 42 active components were associated with 41 common targets. This suggests that multiple compounds within Zhigancao Decoction may act on multiple disease-related targets in a coordinated manner.

Overall, these findings indicate that Zhigancao Decoction may exert therapeutic effects on insomnia–arrhythmia comorbidity through a multi-component, multi-target regulatory mechanism.

### 3.3. Construction of PPI network and screening of core key targets

**Figure 3.**
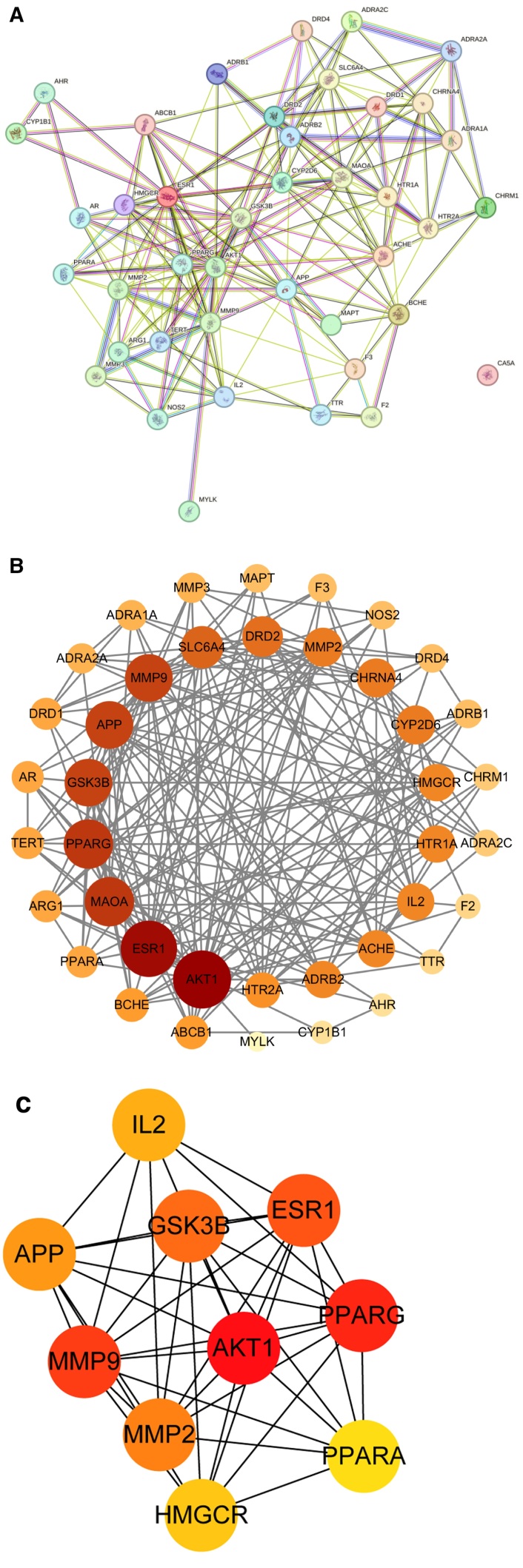
Intersection target PPI network and topology analysis. (A) PPI network diagram. (B) PPI network based on degree value ranking. (C) The 10 core targets selected by the CytoHubba plugin.

The 41 intersection targets of Zhigancao Decoction (ZGCT) in the treatment of insomnia and arrhythmia were imported into the STRING 12.0 database to construct a protein–protein interaction (PPI) network. After removing isolated nodes, the resulting network contained 40 nodes and 197 edges (Fig. 3A).

The network was then imported into Cytoscape 3.9.1 software for visualization and topological analysis. Node degree was used to evaluate the importance of each target in the network, where larger and darker nodes indicated higher centrality (Fig. 3b).

Core targets were further identified using the CytoHubba plugin in Cytoscape 3.9.1, with the MCC (Maximal Clique Centrality) algorithm set to select the top 10 ranked nodes. The identified hub genes included AKT1, PPARG, MMP9, ESR1, GSK3B, MMP2, APP, IL2, HMGCR, and PPARA, which were considered potential key targets for the therapeutic effects of ZGCT in insomnia–arrhythmia comorbidity (Fig. 3C).

### 3.4. Functional enrichment analysis

**Figure 4.**
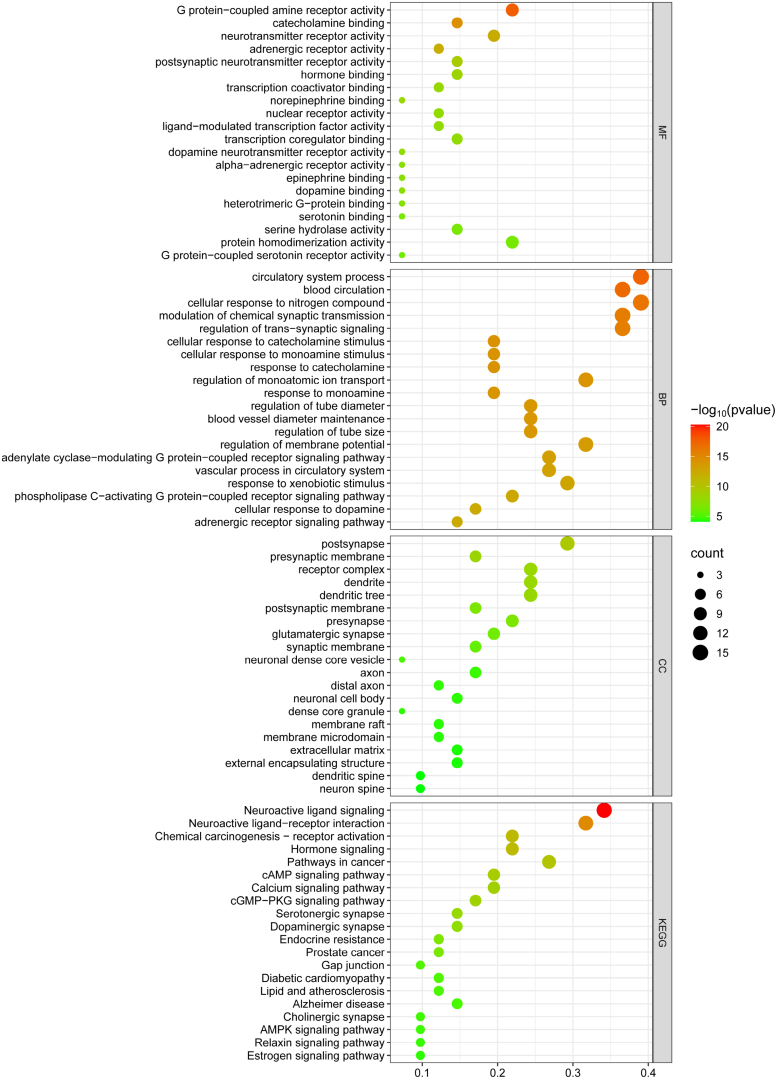
Top 20 enrichment results of GO biological process (BP), cellular component (CC), molecular function (MF), and KEGG pathways for the intersecting targets of Zhigancao Decoction in treating insomnia with palpitations.

To explore the potential mechanisms of Zhigancao Decoction in treating insomnia accompanied by palpitations, GO functional enrichment analysis and KEGG pathway analysis were performed based on 41 intersecting targets. The top 20 enriched GO and KEGG terms were visualized in an integrated enrichment plot (Fig. 4).

GO biological process analysis indicated that the intersecting targets were mainly involved in circulatory system processes, blood circulation, regulation of chemical synaptic transmission, regulation of membrane potential, and ion transport regulation. In addition, processes related to monoamine neurotransmitter response, catecholamine stimulus response, and circadian rhythm regulation were significantly enriched, suggesting potential involvement in autonomic nervous system regulation and sleep–wake cycle control. Overall, GO-BP results suggest that Zhigancao Decoction may exert therapeutic effects through regulation of autonomic activity, neurotransmitter signaling, and cardiovascular functional stability.

GO cellular component analysis revealed that the target genes were mainly localized in synaptic and membrane-related structures, including postsynaptic membrane, presynaptic membrane, synapse, dendritic tree, and receptor complex, indicating that these targets are primarily involved in neuronal signal transmission and synaptic communication processes.

GO molecular function analysis showed that the intersecting targets were mainly associated with G protein-coupled receptor activity, neurotransmitter receptor activity, calcium ion binding, kinase regulatory activity, and transcription co-activator binding, suggesting that the pharmacological effects of Zhigancao Decoction may be mediated through receptor-dependent signaling and intracellular signal transduction pathways.

KEGG pathway enrichment analysis revealed that the intersecting targets were significantly enriched in neuroactive ligand–receptor interaction, serotonergic synapse, dopaminergic synapse, and cholinergic synapse pathways. In addition, cAMP signaling pathway, calcium signaling pathway, and cGMP–PKG signaling pathway were also significantly enriched, indicating involvement in second messenger systems regulating neuronal excitability and cardiomyocyte function. The adrenergic signaling pathway in cardiomyocytes further suggested a role of autonomic nervous system regulation in the therapeutic mechanism.

In addition to these neuroactive and cardiovascular-related pathways, several pathways within the top 20 KEGG results were less directly related to insomnia–palpitation comorbidity, including cancer-related pathways, chemical carcinogenesis–receptor activation, and other broad disease-associated pathways. These pathways are commonly observed in network pharmacology analyses due to the pleiotropic nature of the target genes and may reflect shared biological processes such as inflammation, oxidative stress, and metabolic regulation rather than disease-specific mechanisms. Therefore, subsequent interpretation focused on neuroactive signaling pathways, second messenger systems, and cardiovascular regulatory pathways.

Overall, GO and KEGG enrichment analyses indicate that Zhigancao Decoction may exert therapeutic effects through a multi-target regulatory network involving neurotransmitter signaling, synaptic transmission, second messenger systems, and cardiovascular regulation.

**Figure 5.**
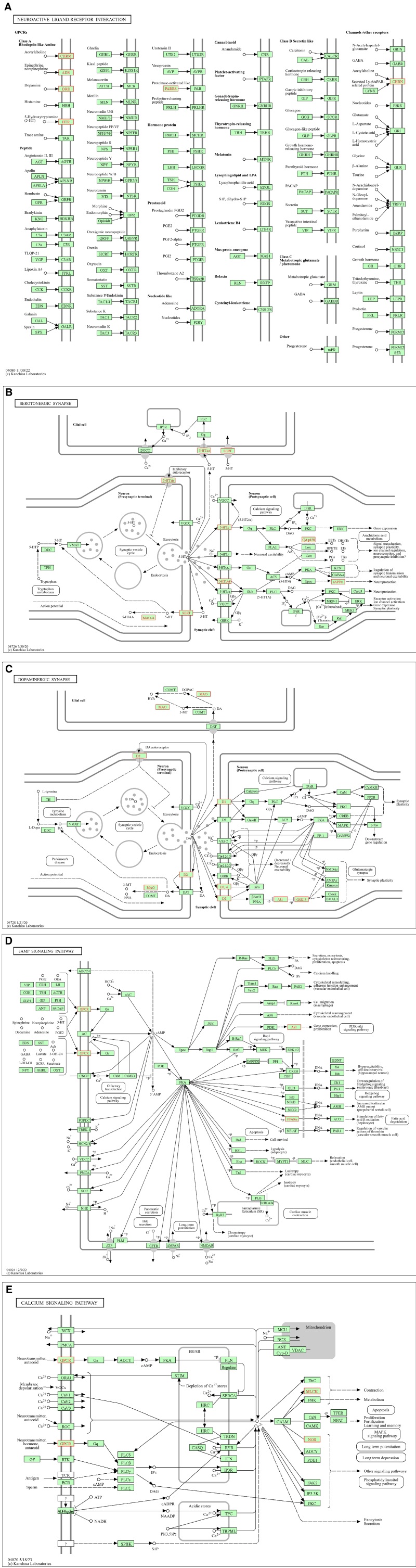
(A) Neuroactive ligand–receptor interaction. (B) Serotonergic synapse. (C) Dopaminergic synapse. (D) cAMP signaling pathway. (E) Calcium signaling pathway. The pathway maps are reproduced from the KEGG database (Copyright Kanehisa Laboratories).

To further validate the KEGG enrichment results, representative pathway maps were constructed for the most significantly enriched signaling pathways, including the neuroactive ligand–receptor interaction pathway, serotonergic synapse, dopaminergic synapse, cAMP signaling pathway, and calcium signaling pathway (Fig. 5).

### 3.5. Molecular docking

Molecular docking analysis was conducted using a target-centered reverse screening framework between the top 10 hub targets and SwissTargetPrediction-derived bioactive compounds.

The full-matrix docking strategy generated a comprehensive ligand–target interaction profile, covering all possible combinations between selected compounds and core targets. The results showed that the generally favorable binding energies observed across the docking matrix suggest that the selected ligand pool may be enriched for compounds with stable binding propensity toward disease-related targets, supporting its role as a structural refinement step in candidate compound screening.

Most ligand–target pairs exhibited binding energies lower than − 5.0 kcal/mol, indicating favorable structural compatibility within receptor binding pockets. This suggests that the docking results primarily reflect structural and energetic compatibility between ligands and target proteins within a multi-component system.

**Figure 6.**
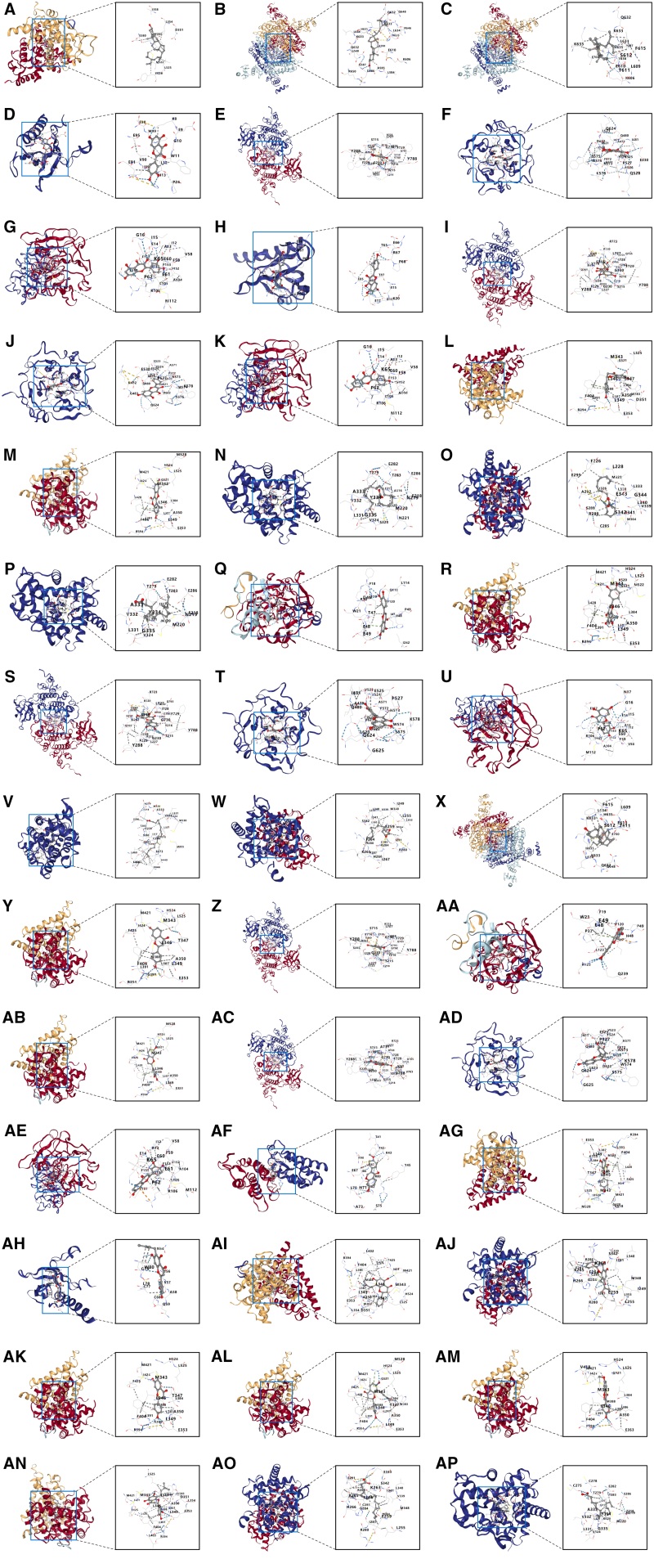
MoIecuIar docking diagram. (A) A2_ESR1; (B) A2_HMGCR; (C) A3_HMGCR; (D) A6_AKT1; (E) A6_GSK3B; (F) A6_MMP2; (G) A6_MMP9; (H) A8_AKT1; (I) A8_GSK3B; (J) A8_MMP2; (K) A8_MMP9; (L) A9_ESR1; (M) DZ1_ESR1; (N) HMR1_PPARA; (O) HMR1_PPARG; (P) HMR2_PPARA; (Q) HMR3_APP; (R) HMR3_ESR1; (S) HMR3_GSK3B; (T) HMR3_MMP2; (U) HMR3_MMP9) RS1_PPARA; (W) RS1_PPARG; (X) SJ1_HMGCR; (Y) TN4_ESR1; (Z) TN4_GSK3B; (AA) TN5_APP; (AB) TN5_ESR1; (AC) TN5_GSK3B; (AD) TN5_MMP2; (AE) TN5_MMP9; (AF) ZGC4_IL2; (AG) ZGC5_ESR1; (AH) ZGC12_AKT1; (AI) ZGC14_ESR1; (AJ) ZGC14_PPARG; (AK) ZGC17_ESR1; (AL) ZGC18_ESR1; (AM) ZGC20_ESR1; (AN) ZGC24_ESR1; (AO) ZGC25_PPARG; (AP) ZHC25_PPARA.

Overall, the docking analysis provides a systematic evaluation of ligand–target interaction potential and serves as a refinement step for narrowing down candidate compounds in network pharmacology-based multi-target studies. The results are summarized in Figure 6 and Table 1.

**Table 1:** Molecular docking results (calculated by binding energy, kcal/mol).

		AKT1	PPARG	MMP9	ESR1	GSK3B	MMP2	APP	IL2	HMGCR	PPARA
A2	Stigmasterol (HCXVJBMSMIARIN-PHZDYDNGSA-N)	−6.6	−10.4	−7.2	*−7.9	−9.6	−7.4	−8.4	−7.4	*−8.1	−8.8
A3	Beta-sitosterol (KZJWDPNRJALLNS-VJSFXXLFSA-N)	−6.5	−10.3	−7.0	−7.4	−8.6	−7.0	−8.0	−8.6	*−8.1	−7.1
A6	Quercetin (REFJWTPEDVJJIY-UHFFFAOYSA-N)	*−6.0	−8.5	*−8.2	−7.7	*−9.9	*−8.7	−7.4	−7.9	−8.4	−8.5
A8	Kaempferol (IYRMWMYZSQPJKC-UHFFFAOYSA-N)	*−6.1	−8.4	*−7.8	−8.3	*−9.3	*−8.8	−7.2	−7.8	−8.1	−8.6
A9	DFV (FURUXTVZLHCCNA-AWEZNQCLSA-N)	−6.0	−7.7	−7.4	*−8.4	−8.7	−7.7	−7.4	−6.4	−8.0	−7.7
DZ1	Coumestrol (ZZIALNLLNHEQPJ-UHFFFAOYSA-N)	−6.5	−8.8	−6.9	*−9.2	−10.1	−8.5	−7.8	−7.8	−7.8	−9.1
HMR1	Arachidonic acid (YZXBAPSDXZZRGB-DOFZRALJSA-N)	−4.9	*−7.3	−5.1	−7.3	−7.2	−6.0	−6.1	−5.6	−5.8	*−7.1
HMR2	Gondoic acid (BITHHVVYSMSWAG-KTKRTIGZSA-N)	−4.3	−6.6	−5.4	−6.9	−6.8	−5.7	−5.4	−5.1	−5.3	*−6.4
HMR3	Luteolin (IQPNAANSBPBGFQ-UHFFFAOYSA-N)	−6.1	−8.6	*−8.0	*−7.9	*−9.6	*−9.0	*−7.6	−8.5	−8.3	−8.3
RS1	5,8,11,14-Eicosatetraenoic acid (YZXBAPSDXZZRGB-CGRWFSSPSA-N)	−4.6	*−7.0	−5.7	−7.4	−7.0	−6.2	−6.1	−6.3	−6.0	*−6.6
SJ1	Gamma-Sitosterol (KZJWDPNRJALLNS-FBZNIEFRSA-N)	−6.8	−9.8	−7.0	−7.1	−8.9	−7.5	−7.9	−7.2	*−8.3	−7.3
TN4	7,4’-Dihydroxyflavone (LCAWNFIFMLXZPQ-UHFFFAOYSA-N)	−6.2	−8.3	−7.6	*−8.4	*−9.2	−9.0	−7.1	−8.5	−8.0	−8.6
TN5	3’,4’,7-Trihydroxyflavone (PVFGJHYLIHMCQD-UHFFFAOYSA-N)	−6.0	−8.6	*−7.9	*−8.2	*−9.5	*−8.8	*−7.4	−8.6	−8.4	−8.4
ZGC4	Formononetin (HKQYGTCOTHHOMP-UHFFFAOYSA-N)	−6.2	−8.3	−7.5	−8.1	−8.6	−7.3	−7.2	*−8.4	−7.8	−8.3
ZGC5	Naringenin (FTVWIRXFELQLPI-ZDUSSCGKSA-N)	−6.3	−8.2	−7.5	*−8.5	−9.0	−8.7	−7.4	−8.4	−8.1	−8.6
ZGC12	Gancaonin O (AFJYQKPCJLMHCC-UHFFFAOYSA-N)	*−6.5	−9.3	−7.7	−7.7	−9.3	−8.7	−8.1	−8.2	−8.7	−8.9
ZGC14	Glabranin (DAWSYIQAGQMLFS-SFHVURJKSA-N)	−6.1	*−9.8	−8.0	*−8.6	−8.7	−8.3	−8.3	−8.3	−8.3	−9.4
ZGC17	(2R)-7-hydroxy-2-(4-hydroxyphenyl) chroman-4-one (FURUXTVZLHCCNA-CQSZACIVSA-N)	−5.9	−8.1	−7.4	*−8.4	−8.8	−8.9	−7.1	−8.6	−7.9	−8.3
ZGC18	(2S)-7-hydroxy-2-(4-hydroxyphenyl)-8-(3-methylbut-2-enyl) chroman-4-one (KYFBXCHUXFKMGQ-IBGZPJMESA-N)	−6.2	−9.1	−7.3	*−9.2	−8.7	−8.7	−8.6	−10.4	−8.5	−9.7
ZGC20	HMO (LNIQZRIHAMVRJA-UHFFFAOYSA-N)	−6.3	−8.2	−7.4	*−8.1	−8.2	−8.5	−7.1	−8.4	−7.8	−8.5
ZGC24	8-prenylated eriodictyol (XJUDJFVOICLOMY-GOSISDBHSA-N)	−6.5	−8.8	−6.8	*−8.8	−8.2	−8.2	−8.5	−7.6	−8.4	−7.9
ZGC25	Gadelaidic acid (LQJBNNIYVWPHFW-VAWYXSNFSA-N)	−4.4	*−6.3	−5.4	−7.0	−6.3	−5.5	−5.9	−5.0	−5.7	*−6.3

* indicates high-confidence targets predicted by SwissTargetPrediction (*p* > .3).

## 4. Discussion

### 4.1. Mechanistic interpretation of insomnia–arrhythmia comorbidity

This study constructed a systems-level network pharmacology and molecular docking framework to elucidate the mechanisms underlying insomnia–arrhythmia comorbidity and the action profile of Zhigancao Decoction (ZGCT). The results suggest that insomnia-related chronic stress may contribute to arrhythmia susceptibility through a multi-level regulatory network involving neuroendocrine activation, autonomic nervous system imbalance, inflammatory signaling, and cardiovascular remodeling.

Chronic sleep disturbance is closely associated with activation of the hypothalamic–pituitary–adrenal (HPA) axis and sympathetic nervous system, accompanied by systemic inflammation and oxidative stress. These upstream alterations may disrupt cardiovascular homeostasis and create a pro-arrhythmic biological environment.

A central regulatory axis identified in this study is the renin–angiotensin system (RAS), characterized by imbalance between the classical ACE/Ang II/AT1R pathway and the nonclassical ACE2/Ang-(1–7)/Mas receptor pathway. Such imbalance may promote vasoconstriction, inflammatory activation, and fibrotic signaling, thereby contributing to myocardial electrical and structural remodeling.

Importantly, recent evidence suggests that nonclassical RAS components may interact with intracellular signaling pathways, including AKT-related signaling cascades, thereby linking neurohormonal dysregulation with downstream cellular responses involved in cardiovascular remodeling.^[24]^ This provides a plausible mechanistic connection between systemic neuroendocrine stress and intracellular signaling alterations.

Downstream of RAS-related signaling, key effector targets identified in this study include AKT1, PPARG, and MMP9. AKT1 is involved in intracellular signal transduction and may contribute to electrophysiological regulation and cellular survival processes. PPARG is associated with metabolic and inflammatory regulation, suggesting a potential role of metabolic-inflammatory coupling in disease progression. MMP9 is a key regulator of extracellular matrix remodeling and may contribute to structural changes in the atrial myocardium.

Collectively, these molecular alterations may contribute to atrial electrical instability, ion channel dysfunction, and structural fibrosis, thereby increasing susceptibility to arrhythmia development.

### 4.2. Chemical characteristics of the prioritized candidate compounds

The target-centered reverse screening strategy identified 22 candidate bioactive compounds, which can be broadly classified into 3 major chemical categories: phytosterols, flavonoids, and long-chain unsaturated fatty acids. Specifically, the phytosterol group comprised stigmasterol, β-sitosterol, and γ-sitosterol; the flavonoid group included quercetin, kaempferol, DFV, coumestrol, luteolin, 7,4′-dihydroxyflavone, 3′,4′,7-trihydroxyflavone, formononetin, naringenin, Gancaonin O, glabranin, (2R)-7-hydroxy-2-(4-hydroxyphenyl) chroman-4-one, (2S)-7-hydroxy-2-(4-hydroxyphenyl)-8-(3-methylbut-2-enyl) chroman-4-one, HMO, and 8-prenylated eriodictyol; and the long-chain unsaturated fatty acid group consisted of arachidonic acid, gondoic acid, 5,8,11,14-eicosatetraenoic acid, and gadelaidic acid. These chemically diverse compounds were identified through the target-centered reverse screening workflow and demonstrated favorable predicted binding affinities toward the prioritized hub proteins.

Among these candidates, flavonoids represented the largest proportion of the prioritized compounds and generally exhibited favorable docking affinities toward several hub targets, suggesting that they may contribute to the regulation of oxidative stress, inflammatory signaling, and neurocardiovascular function. In comparison, phytosterols showed relatively stable interactions with lipid metabolism- and inflammation-related targets such as PPARG and PPARA, whereas the long-chain unsaturated fatty acids displayed moderate but consistent binding profiles across multiple targets, indicating that they may provide complementary biological activities rather than acting as dominant active constituents. Collectively, these findings support the concept that the therapeutic effects of Zhigancao Decoction are likely mediated by the coordinated actions of structurally diverse compounds on multiple disease-related targets, which is consistent with the multi-component and multi-target characteristics of traditional Chinese medicine.

### 4.3. Clinical implications: atrial fibrillation and catheter ablation

Increasing clinical evidence suggests that sleep disorders are not only associated with the development of atrial fibrillation but may also contribute to treatment-resistant or recurrent arrhythmias, particularly in elderly individuals and patients with multiple comorbidities.^[2,3]^ Persistent sleep fragmentation may induce chronic sympathetic activation, autonomic imbalance, systemic inflammation, and neuroendocrine dysregulation, thereby promoting atrial electrical and structural remodeling and increasing susceptibility to arrhythmia recurrence.^[6,7]^ Therefore, effective management of sleep disturbances may represent an important adjunctive strategy for improving long-term rhythm-control outcomes in patients with atrial fibrillation.^[3,10]^

From a clinical perspective, pulmonary vein isolation (PVI) remains the cornerstone of catheter ablation for atrial fibrillation (AF). However, recurrence of atrial arrhythmia may still occur even in patients with durable PVI, indicating that AF maintenance is not solely dependent on pulmonary vein triggers.

Against this clinical background, recent studies have shown that patients with arrhythmia recurrence after durable PVI may benefit from more comprehensive ablation strategies, including atrial substrate modification, low-voltage area ablation, and non-pulmonary vein trigger ablation, rather than PVI alone.^[25]^

In addition, meta-analytic evidence has demonstrated that catheter ablation in AF patients with heart failure significantly reduces all-cause mortality, improves left ventricular function, and decreases AF recurrence without increasing adverse events.^[26]^

These findings suggest that residual arrhythmia risk after ablation is multifactorial and may involve both electrophysiological triggers and structural substrate abnormalities. In this context, sleep-related autonomic imbalance and inflammatory activation may further increase atrial vulnerability and potentially reduce long-term ablation efficacy.

Therefore, AF recurrence after PVI should be considered a complex process involving systemic neurocardiovascular dysregulation rather than a purely procedural limitation. These observations further support the rationale for complementary multi-target therapeutic strategies aimed at improving the neurocardiovascular environment in addition to conventional rhythm-control interventions.

### 4.4. Methodological innovation: target-centered reverse screening strategy

Traditional network pharmacology approaches often rely on component-centered ranking based on topological parameters such as degree centrality, which may lead to redundancy and limited target specificity. In contrast, this study adopts a target-centered reverse screening strategy, in which disease-relevant hubproteins are first identified, followed by computational prediction of candidate bioactive compounds based on SwissTargetPrediction and molecular docking-derived interaction scores.

It should be emphasized that this approach does not imply experimentally validated or specific molecular binding, but rather provides computational estimates of potential interaction likelihood and structural complementarity. This strategy reduces noise from nonspecific multi-target predictions and allows prioritization of candidate compounds for downstream experimental validation.

### 4.5. Overall interpretation and future perspectives

Collectively, this study proposes a convergent systems-level framework linking insomnia-related neuroendocrine activation, renin–angiotensin system (RAS) imbalance, and downstream signaling cascades to arrhythmogenesis, suggesting a coordinated brain–heart regulatory network underlying insomnia–arrhythmia comorbidity.

Although these findings are based on computational predictions and remain hypothesis-generating, they provide a structured mechanistic basis for prioritizing candidate targets and bioactive compounds in Zhigancao Decoction.

Future studies incorporating electrophysiological experiments, animal models, and clinical validation will be essential to verify the biological relevance and causal roles of the predicted targets and pathways.

**Figure 7.**
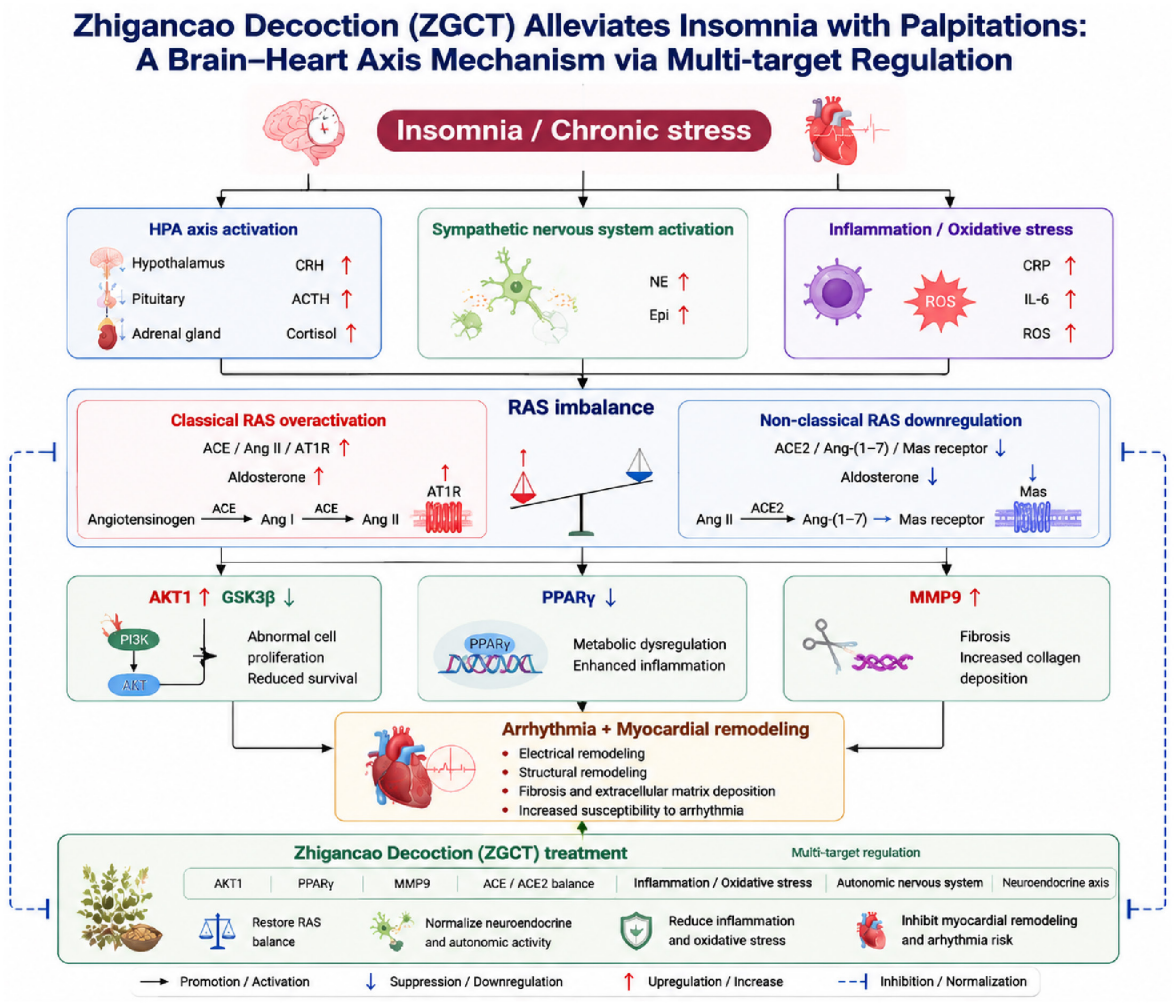
Integrated brain–heart axis mechanism of Zhigancao Decoction in insomnia–arrhythmia comorbidity.

The integrated brain–heart axis mechanism of Zhigancao Decoction is illustrated in Figure 7.

## 5. Conclusion

In this study, a network pharmacology and molecular docking-based framework was employed to investigate the potential mechanisms of Zhigancao Decoction in the treatment of insomnia–arrhythmia comorbidity. The results suggest that the therapeutic effects of Zhigancao Decoction may be associated with multi-target regulation involving neuroendocrine regulation, renin–angiotensin system signaling, and cardiovascular remodeling.

Importantly, a target-centered reverse screening strategy was proposed, enabling the prioritization of disease-relevant hub proteins and the identification of candidate bioactive compounds with potential interaction affinity. This approach provides a more focused and interpretable compound screening strategy compared with conventional component-centered network pharmacology methods.

Although the findings are based on computational predictions and require further experimental validation, they provide a systems-level hypothesis for brain–heart axis interactions and may inform future multi-target therapeutic development for complex comorbid conditions.

## Acknowledgments

We much appreciate College of Traditional Chinese Medicine of Beijing University of Chinese Medicine for providing support on the study.

## Author contributions

**Conceptualization:** Bolun Xue.

**Data curation:** Bolun Xue.

**Formal analysis:** Bolun Xue.

**Investigation:** Bolun Xue.

**Methodology:** Bolun Xue.

**Project administration:** Bolun Xue.

**Resources:** Bolun Xue.

**Software:** Bolun Xue.

**Supervision:** Bolun Xue.

**Validation:** Bolun Xue.

**Visualization:** Bolun Xue.

**Writing – original draft:** Bolun Xue

**Writing – review & editing:** Bolun Xue, Linghui Lu, Wei Wang..

**Funding acquisition:** Yong Wang.
